# A Prospective Study Assessing Functional Outcomes of Arthroscopic Subacromial Decompression with Platelet-Rich Plasma Augmentation in Shoulder Impingement Syndrome

**DOI:** 10.5704/MOJ.2511.015

**Published:** 2025-11

**Authors:** V Kumar, V Khatkar, R Potalia, PK Majumdar, A Vashisth, V Dewhari, S Tibrewal

**Affiliations:** Department of Orthopaedics, Pandit Bhagwat Dayal Sharma Post Graduate Institute of Medical Sciences, Rohtak, India

**Keywords:** shoulder impingement syndrome, platelet-rich plasma, arthroscopic subacromial decompression

## Abstract

**Introduction:**

Shoulder impingement syndrome (SIS) arises from inflammation and degeneration of structures in subacromial space, significantly affects daily functioning, and is a frequent reason for primary care consultations. Extrinsic factors include pathological anatomical variations in the acromion and thickening of coracoacromial ligament, along with Intrinsic factors resulting from aging, inadequate blood supply, genetic predisposition, occupation, or lifestyle-induced stress.

**Materials and methods:**

This study, uniquely designed to assess the effectiveness of a consensus of clinical guidelines on the effectiveness of arthroscopic subacromial decompression (ASD) with intra-operative platelet-rich plasma (PRP) augmentation in SIS, analysed seventy-two patients. Their functional outcomes were determined using Visual Analogue Scale, Constant Shoulder Score, and UCLA Score.

**Results:**

The findings revealed significant improvements across all outcome measures. The most striking was the substantial pain reduction, as evidenced by the mean VAS score decreasing from 5.88 pre-operatively to 1.08. This significant reduction in pain offers substantial hope and optimism for patients suffering from SIS. The constant scores increased from 38.04 to 82.71, with excellent or good results in 91.67% of the patients. The UCLA scores increased from 14.50 to 31.13, with 91.67% of patients having excellent or good results. Importantly, no significant complications were observed.

**Conclusion:**

Arthroscopic subacromial decompression, augmented with PRP, is a safe and effective procedure in treating SIS, yielding significant improvements in clinical outcomes (pain and function) by the present study. The functional result is comparable for patients with intact and partial rotator cuff tears. This study's findings and safety profile reiterate the effectiveness of this procedure, instilling confidence in the practitioner regarding its application.

## Introduction

Shoulder impingement syndrome (SIS) is a common musculoskeletal condition characterised by inflammation and deterioration of structures within the subacromial space that leads to the impingement of the rotator cuff together with the subacromial bursa structures^1^. Changes within the subacromial space may worsen impingement during shoulder movements with 90° abduction combined with 45° internal rotation^2^. Neer divided SIS into three phases, ranging from simple reversible edema and haemorrhage in younger patients to degenerative tendinopathy, rotator cuff tears, and osseous changes in older individuals^3^. SIS aetiology incorporates extrinsic and intrinsic factors in its origin. Extrinsic factors encompass pathological anatomical variations in the acromion, thickening of coracoacromial ligament, subacromial bursitis, and abnormal posture^4^. Intrinsic factors are related to tendon degeneration resulting from aging, inadequate blood supply, genetic predisposition, occupation, or lifestyle-induced stress^5^. SIS significantly affects daily functioning and is a frequent reason for primary care consultations, with incidence rates increasing with age^6^. Non-operative and surgical approaches exist for the treatment of SIS. Exercise programs, Corticosteroid treatment, NSAIDs, Physical therapy, and taping are all identified conservative treatments that tend to work^7^. Arthroscopic subacromial decompression alleviates impingement by increasing the subacromial space and excising the pathological tissue. This procedure is performed on patients who do not respond to conservative treatment approaches^8^.

Platelet-rich plasma (PRP) has emerged as a potential biological therapeutic agent for rotator cuff tendinopathy in recent years. PRP is an autologous concentration of platelets containing growth factors, such as platelet-derived growth factors (PDGFs), transforming growth factors (TGFs), vascular endothelial growth factor (VEGF), and epithelial growth factor (EGF), which may enhance tendon healing^9,10^. Although the use of PRP has increased in clinical practice, very few randomised control studies have examined its usefulness in the non-operative care of rotator cuff tendinopathy^11^. Considering the potential benefits of PRP and arthroscopic subacromial decompression, this study investigated their combined impact on functional outcomes in patients with SIS.

## Materials and Methods

The study group included patients suffering from shoulder pain lasting for at least three months with SIS confirmed through MRI scans. The patients' demographics were diverse, with a range of ages and occupations, reflecting the real-world population affected by SIS. The exclusion criteria comprised radiating pain, adhesive capsulitis, calcific tendinitis, biceps tendon displacement, superior labrum anterior-posterior (SLAP) lesion, recent shoulder operation within six months, systemic connective tissue disorders, complete rotator cuff tear visible on MRI, shoulder joint laxity or instability, recent corticosteroid injection within three months, and blood disorders or coagulation abnormalities. Based on previous research on the functional outcomes of arthroscopic subacromial decompression and autologous platelet-rich plasma (PRP) in SIS, the study assumed a 78% treatment efficacy, with a 10% error margin and 5% significance level. In an attempt to increase the validity of the research, 75 patients were enrolled, of whom only 72 were evaluated due to loss of follow-up and were assessed for functional outcomes. The control group was excluded because the study was prospective and real-world, concentrating on functional improvements. Ethical concerns about withholding treatment from surgical patients also influenced the decision. Finally, historical benchmarks offered indirect comparison points.

PRP Preparation: PRP was prepared using a standardised protocol on the day of surgery. Thirty millilitres of venous blood were extracted and mixed with citrate phosphate dextrose adenine (CPDA) at a 1:9 ratio. The blood was centrifuged at 2000 RPM for 10 min and separated into three layers: erythrocytes (bottom), platelet layer (middle), and platelet-poor plasma (PPP, top). After removing the red blood cells, the remaining sample was centrifuged at 2000 RPM for 10 min. Approximately 75% of PPP was discarded, leaving 6mL of PRP, then buffered with sodium bicarbonate (0.5mL)^12^. The PRP was injected intra-operatively using a 25-gauge spinal needle.

All patients underwent arthroscopic subacromial decompression under general anaesthesia in the lateral decubitus position. The operative arm was secured in a foam traction sleeve attached to a traction device with 40 N of tension. The shoulder was positioned at 10° – 20° flexion and 40° abduction. Bone landmarks, including the acromion, clavicle, acromioclavicular joint, coracoid process, and coracoacromial ligament, were marked. A standard posterior portal was created for arthroscopic visualisation of the glenohumeral and subacromial spaces. A lateral portal was used for the instrumentation. Subacromial decompression involved debridement of the subacromial bursa, resection of the anterolateral acromion and underhanging osteophytes from the acromioclavicular joint using a shaver blade and burr, and haemostasis using radiofrequency ablation cautery. This process effectively increases the subacromial space and alleviates impingement. Post-decompression PRP was injected into the subacromial space via the lateral portal under arthroscopic visualisation.

Antibiotics (Ceftriaxone, Amikacin) were administered perioperatively. Initially, a supportive sling was given postoperatively. Analgesic medications (Tramadol and Paracetamol) were administered as required. The sutures were removed two weeks after the procedure. To prevent adhesions, shoulder rehabilitation exercises were commenced concurrently with passive shoulder movement on the first post-operative day. Active exercises were performed after suture removal. Three weeks after surgery, exercises to strengthen the rotator cuff were initiated. Additionally, patients with partial-thickness rotator cuff tears underwent arthroscopic subacromial decompression (ASD) with intra-operative platelet-rich plasma (PRP) augmentation, without further repair. The aim was to evaluate the outcomes of ASD with PRP across various tear severities. In accordance with guidelines, non-retractable partial tears and stable shoulders were left untreated with sutures.

Following surgery, patients were monitored at three weeks, three months, and six months. During these follow-up visits, clinicians documented cutaneous discoloration, pain, infection, or other complications. The efficacy of the treatment was evaluated using the visual analogue scale (VAS), Constant Shoulder Score, and UCLA Score at each follow-up appointment: three weeks, three months, and six months post-operatively. The collected data were analysed to assess functional outcomes.

Standard statistical methods were used to examine all measurements and data. Variables with a normal distribution were expressed as means and standard deviations. Analysis was conducted using Student’s t-test for data following a normal distribution. Categorical and ordinal data were evaluated using the chi-squared test.

## Results

This study included 72 patients diagnosed with shoulder impingement syndrome. The mean age was 51.88 years, with 54.17% of the patients exceeding 50. A slight female predominance was observed (58.33% female vs 41.67% male). The right (dominant) shoulder was affected in 66.67% of cases. The mean duration of pain was 11.46 months, with an equal distribution between patients experiencing pain for less than or more than one year. Type 2 acromion was the most prevalent (62.50%), followed by type 1 (25%) and type 3 (12.50%). The mean acromiohumeral distance on preoperative MRI was 7.43mm, with 75% of patients exhibiting a distance greater than 7mm. Pre-operative MRI revealed partial rotator cuff tears in 45.83% of patients, while 54.17% had intact rotator cuff tears. The most frequent observations on arthroscopy were subacromial bursitis (91.66%), acromial spurs (58.33%), and bursal-side supraspinatus tears (33.33%).

The VAS scores demonstrated a statistically significant improvement from a mean of 5.88 pre-operatively to 1.08 six months post-operatively. Constant shoulder scores improved from a mean of 38.04 pre-operatively to 82.71 six months post-operatively. At the 6-month follow-up, 54.17% of the patients exhibited good results, and 37.50% demonstrated excellent results. UCLA scores increased from a mean of 14.50 pre-operatively to 31.13 six months post-operatively. At the 6-month follow-up, 62.50% of the patients exhibited good results, and 29.17% demonstrated excellent results. The comparative analysis showed comparable results between the left and right shoulders (Table I) or between patients with partial rotator cuff tears and those with intact rotator cuff tears (Table II).

**Table I:** Comparative statistics of VAS score, constant shoulder score, and UCLA score at different time points on the left and right shoulder.

Time Points	Parameters	VAS Score		Constant Shoulder Score		UCLA Score	
		Left Side	Right Side	Left Side	Right Side	Left Side	Right Side
		(N = 24)	(N = 48)	(N = 24)	(N = 48)	(N = 24)	(N = 48)
Pre-Op	Mean	6.13	5.75	37.38	38.38	15.00	14.25
	S.D.	0.83	0.77	5.78	4.54	2.51	2.02
	Maximum Score	7.00	7.00	44.00	48.00	18.00	18.00
	Minimum Score	5.00	5.00	28.00	30.00	10.00	11.00
After 3 Weeks	Mean	2.88	2.81	59.50	61.19	22.75	22.13
	S.D.	0.99	0.54	5.32	6.20	2.31	2.36
	Maximum Score	4.00	4.00	66.00	74.00	26.00	26.00
	Minimum Score	2.00	2.00	52.00	50.00	20.00	18.00
After 3 Months	Mean	1.75	1.75	71.88	71.81	28.13	27.50
	S.D.	0.89	0.58	4.29	6.01	2.53	2.83
	Maximum Score	3.00	3.00	78.00	84.00	32.00	32.00
	Minimum Score	1.00	1.00	66.00	58.00	25.00	21.00
After 6 Months	Mean	1.25	1.00	80.88	83.63	30.63	31.38
	S.D.	1.39	0.63	10.55	6.27	4.21	2.96
	Maximum Score	4.00	2.00	90.00	94.00	35.00	35.00
	Minimum Score	0.00	0.00	60.00	70.00	22.00	24.00

P-value (by one-way Anova-F test) among different time-pointsP = .0122* P < .05 (Sig.Diff.)  P = .0174* P < .05 (Sig.Diff.)  P = .0073* P < .05 (Sig.Diff.)  P = .0027* P < .05 (Sig.Diff.)  P = .0007* P < .05 (Sig.Diff.)  P = .0009* P < .05 (Sig.Diff.)

**Table II:** Comparative statistics of VAS score, constant shoulder score, and UCLA score at different time points in pre-op MRI findings for partial rotator cuff tear and intact rotator cuff category.

Time Points	Parameters	VAS Score		Constant Shoulder Score		UCLA Score	
		Partial Rotator Cuff Tear (N = 33)	Intact Rotator Cuff (N = 39)	Partial Rotator Cuff Tear (N = 33)	Intact Rotator Cuff (N = 39)	Partial Rotator Cuff Tear (N = 33)	Intact Rotator Cuff (N = 39)
Pre-Op	Mean	5.91	5.85	36.64	39.23	14.36	14.62
	S.D.	0.83	0.80	4.63	4.95	1.86	2.47
	Maximum Score	7.00	7.00	43.00	48.00	17.00	18.00
	Minimum Score	5.00	5.00	30.00	28.00	11.00	10.00
After 3 Weeks	Mean	2.91	2.77	59.36	61.69	21.91	22.69
	S.D.	0.70	0.73	4.95	6.54	1.87	2.66
	Maximum Score	4.00	4.00	66.00	74.00	24.00	26.00
	Minimum Score	2.00	2.00	52.00	50.00	19.00	18.00
After 3 Months	Mean	1.91	1.62	71.27	72.31	27.18	28.15
	S.D.	0.54	0.77	3.58	6.69	2.18	3.08
	Maximum Score	3.00	3.00	75.00	84.00	31.00	32.00
	Minimum Score	1.00	1.00	64.00	58.00	24.00	21.00
After 6 Months	Mean	1.36	0.85	80.91	84.23	30.18	31.92
	S.D.	1.03	0.80	7.94	7.70	3.40	3.23
	Maximum Score	4.00	2.00	90.00	94.00	34.00	35.00
	Minimum Score	0.00	0.00	60.00	70.00	22.00	24.00

P-value (by one-way Anova-F test) among different time-pointsP = .0048* P < .05 (Sig.Diff.)  P = .0036* P < .05 (Sig.Diff.)  P = .0085* P < .05 (Sig.Diff.)  P = .0005* P < .05 (Sig.Diff.)  P = .0039* P < .05 (Sig.Diff.)  P = .0152* P < .05 (Sig.Diff.)

Two patients exhibited suboptimal Constant and UCLA scores, and one demonstrated regression improvement between the three and six months of follow-up. No significant complications were observed, such as infection, neurovascular injury, or anaesthesia-related adverse events. This study's findings and the consistently observed safety profile of this procedure should instil confidence in the practitioner regarding its application, providing a sense of security in the face of a challenging condition.

## Discussion

The treatment of shoulder impingement syndrome encompasses both operative and non-operative strategies, and the severity of the condition primarily influences the selection of the treatment modality. However, the efficacy of both surgical and non-surgical approaches in addressing shoulder impingement syndrome remains a subject of debate in the scientific literature. Research indicates that conservative therapies can yield favourable outcomes in many patients, particularly in cases of mild severity. While non-operative conservative treatments, including physiotherapy and anti-inflammatory medications, have proven efficacious for a substantial proportion of patients, surgical intervention may be necessary when these methods fail to alleviate symptoms^8^. Arthroscopic procedures are gaining prominence for treating refractory shoulder impingement owing to their minimally invasive nature, reduced risk of infection, and expedited recovery compared to conventional open surgical techniques.

This study focused on managing shoulder impingement syndrome and evaluated the outcomes of arthroscopic subacromial decompression with intra-operative platelet-rich plasma (PRP) augmentation. The study comprised 75 patients, with three patients lost to follow-up, resulting in 72 patients being evaluated for functional outcomes. The sample size was bigger than those of several other studies in the literature, ranging from 20 to 42 patients^13,14^. The mean age of the patients was 51.88 years (range: 35 – 63 years). The sex distribution exhibited a slight female predominance, with 14 females (58.33%) and 10 males (41.67%). The mean duration of pain was 11.46 months (range: 7 –18 months). This aligns with findings from Lim *et al* (12.4 months, range: 3 – 96 months)^13^, Khare *et al* (12.95 months) ^14^, and Dom *et al* (18.2 months, range: 3 months – 7 years)^15^.

The right shoulder, the dominant side for all patients, was affected in 66.67% of the cases. This finding aligns with Patel *et al* (58% dominant vs 42% non-dominant)^16^, David *et al* (87.5% right vs 12.5% left in the arthroscopic subacromial decompression + PRP group)^17^, and Speer *et al* (71% dominant vs 29% non-dominant)^18^. Regarding acromion types, in the present study of 72 patients, the distribution of acromion types was as follows: 25% of patients had type 1 acromion, 62.50% had type 2 acromion and 12.50% of patients had type 3 acromion. This is consistent with Khanal *et al* (45.7% type 1, 48.6% type 2, 5.7% type 3)^19^ and Dom *et al* (23.1% type 1, 61.53% type 2, 15.3% type 3)^15^. The mean acromiohumeral distance was 7.43mm (range: 6.50 to 8.30mm). Comparison with other studies shows varying preoperative acromiohumeral distances: Lim *et al* reported 7.9mm (range 6–12mm)^13^, Khanal *et al* found 7.43mm in impingement patients^19^, and Siron *et al* observed 9.77mm, with 16 patients ≥ 10mm, 11 between 7 – 9.9mm, and 1 < 7mm^20^. Of the 72 patients, 54.17% had an intact rotator cuff, and 45.83% had a partial rotator cuff tear. The study found no statistically significant difference in functional outcomes between patients with intact and partial rotator cuff tears. Siron *et al* studied 28 patients, finding 57% with intact rotator cuffs and 43% with partial tears, with no significant difference in Constant scores at 6 months (87.81 vs 87.0)^20^. Rehman *et al* studied 30 patients (77% intact, 23% partial tears) and found a significant score difference, with intact cuffs improving from 50 to 89 and partial tears from 39 to 83 post-op^21^.

**Fig. 1:**
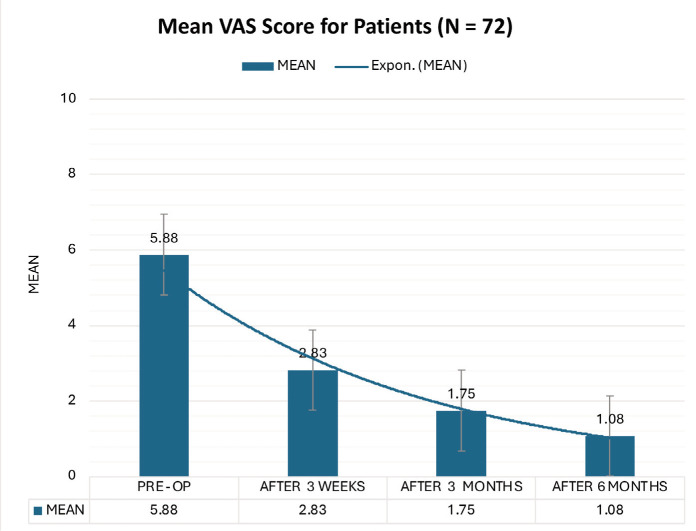
Bar diagram of average VAS score of all patients at different time points.

**Fig. 2:**
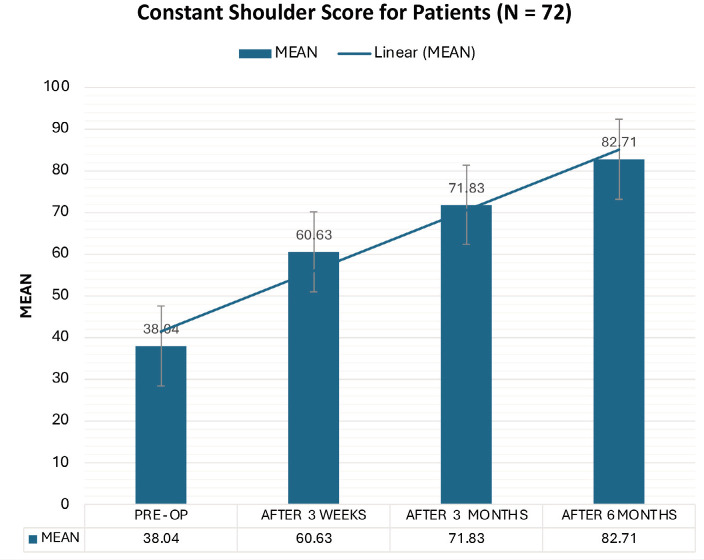
Bar diagram of average constant shoulder score of all patients at different time points.

**Fig. 3:**
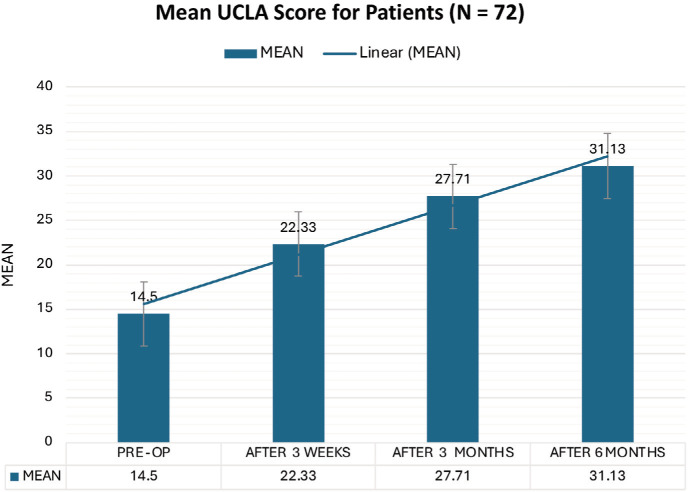
Bar diagram of the average UCLA score of all patients at different time points.

This study used three primary outcome measures to assess patient improvement. The Visual Analog Scale (VAS) for pain significantly reduced, with the mean pre-operative score of 5.88 decreasing to 1.08 at six months post-surgery (Fig. 1). This finding is consistent with the results reported by Lunsjo *et al*, who observed a significant improvement in VAS scores from 7 to 1 at 6 months^22^. Similarly, David *et al* documented a 3-point reduction (from 4.16 to 1.17) in the arthroscopic subacromial decompression with the PRP group^17^. Jaiswal *et al* noted a decrease from 9.03 to 0.48 following arthroscopic subacromial decompression^23^. The Constant Shoulder Score improved from a mean of 38.04 pre-operatively to 82.71 at six months, with 91.67% of patients achieving excellent-to-good scores (Fig. 2). This finding is consistent with the results reported by Rehman *et al*, who observed a mean Constant score improvement from 48 pre-operatively to 88 at six months post-operatively^21^. David *et al* documented an increase from 38 to 79 in ASD+PRP patients^17^. At the same time, Jarvela *et al* noted an elevation from 60 to 92 in hospitalised patients^24^. Similarly, the UCLA Shoulder Score increased from a pre-operative mean of 14.5 to 31.33 at six months, where 37.50% of patients attained excellent scores, 54.17% had good scores, and 8.33% had fair scores (Fig. 3). These results are comparable with those reported by Samanta *et al*, who observed a mean UCLA score improvement from 12.66 pre-operatively to 29.14 at 16 weeks^25^. Similarly, Ravi Kiran *et al* documented scores increasing from 8.25 to 29.00 over 12 months^26^. Jaiswal *et al* noted an increase from 9.97 to 30.9 following arthroscopic subacromial decompression^23^. Jarvela *et al* reported an elevation from 19 to 32 in hospitalised patients^24^, while Aydin *et al* observed an improvement from 7.86 to 30.61 post-operatively^27^.

None of the patients experienced an infection, inflammation, or shoulder stiffness. However, six patients demonstrated fair results for both Constant and UCLA scores. Three patients experienced loss of improvement, potentially due to acromioclavicular joint arthritis, inadequate resection, or recurrence of subacromial bursitis. One fascinating insight from this study is the lack of statistically significant differences in functional outcomes between patients with intact rotator cuffs and those with partial tears. This suggests that a partial rotator cuff tear may not necessarily predict poorer outcomes following arthroscopic subacromial decompression with PRP augmentation. Another noteworthy observation is the consistent improvement across all assessment tools (VAS, Constant, and UCLA scores) over the six months, indicating the procedure's effectiveness in managing shoulder impingement syndrome. The study also highlights the importance of long-term follow-up, as some patients may experience a loss of improvement over time, possibly due to factors such as acromioclavicular joint arthritis or recurrence of subacromial bursitis.

The study's innovative approach of combining ASD with PRP sets it apart from previous research and provides valuable insights into SIS treatment. Nonetheless, it has limitations, including the necessity for long-term follow-up and larger participant groups to validate the results, which could be influenced by recall bias. Future studies might include platelet quantification to examine the dose-response effect and verify PRP's biological activity in shoulder impingement syndrome. Significantly, this is the first research involving the South Asian/Indian population.

## Conclusion

The study concluded that arthroscopic subacromial decompression with intra-operative PRP augmentation was beneficial in patients with shoulder impingement who failed conservative treatment. The unique aspect of our research lies in its comprehensive approach, which includes using a consensus of clinical guidelines and incorporating PRP augmentation, a promising technique. The procedure significantly improved functional outcomes and was equally effective in patients with an intact rotator cuff or partial tears. The arthroscopic procedure was deemed safe, with minimal complications. The results are comparable to those of other studies in the literature, with good-to-excellent outcomes in most patients. However, the optimal dose and activation method for PRP remains uncertain, limiting the ability to draw a definitive conclusion.
